# Case Report: Anti-GABA-B receptor encephalitis with refractory status epilepticus and favorable short-term recovery

**DOI:** 10.3389/fimmu.2026.1896400

**Published:** 2026-08-04

**Authors:** Min-Jing Chou, Pang-Yen Hsu, Ming-Chen Tsai, Chih-Wei Wang, Fu-Chi Yang

**Affiliations:** 1 Department of Neurology, Tri-Service General Hospital, National Defense Medical University, Taipei, Taiwan; 2 Department of Radiology, Tri-Service General Hospital, National Defense Medical University, Taipei, Taiwan

**Keywords:** anti-GABA-B receptor encephalitis, autoimmune encephalitis, case report, refractory status epilepticus, therapeutic plasma exchange

## Abstract

Anti-gamma-aminobutyric acid type B receptor (anti-GABA-B receptor) encephalitis is a rare autoimmune limbic encephalitis that frequently presents with seizures and may be associated with malignancy, most commonly small-cell lung cancer. We report a 66-year-old man with anti-GABA-B receptor encephalitis who presented with a witnessed generalized tonic–clonic seizure after a 2-day prodrome of nocturnal repetitive stereotyped behaviors retrospectively suspicious for subtle focal seizures with automatisms. Brain magnetic resonance imaging showed right-predominant hippocampal and multifocal cortical hyperintensities, and cerebrospinal fluid analysis showed mild pleocytosis and elevated protein with negative infectious testing. He deteriorated on hospital day 5 with refractory status epilepticus and hypoxemic respiratory failure requiring intubation and intensive care. Anti-GABA-B receptor antibodies were subsequently confirmed in both serum and cerebrospinal fluid. Initial malignancy evaluation included contrast-enhanced computed tomography of the chest, abdomen, and pelvis; serum tumor markers; and an onconeural antibody panel. No malignancy was detected. Treatment included intravenous methylprednisolone, 10 sessions of therapeutic plasma exchange, and multi-agent antiseizure therapy. He was extubated after approximately 2 weeks and discharged on hospital day 39 with a modified Rankin Scale score of 3. At 3 months, he was seizure-free, with a modified Rankin Scale score of 1, a Barthel Index of 95/100, and a Mini-Mental State Examination score of 30/30. This single uncontrolled case is hypothesis-generating and does not establish treatment efficacy, optimal plasma exchange duration, long-term prognosis, or definitive non-paraneoplastic status. It illustrates that new-onset refractory status epilepticus should prompt expedited autoimmune encephalitis evaluation and that structured oncologic surveillance remains necessary despite negative initial tumor screening.

## 1 Introduction

Autoimmune encephalitis is a time-sensitive neurologic emergency in which delayed recognition may worsen outcomes (1, 2). Because antibody results are often unavailable during the initial diagnostic window, contemporary criteria emphasize syndrome-based recognition using clinical semiology, neuroimaging, electroencephalography (EEG), cerebrospinal fluid (CSF) findings, and exclusion of alternative causes (1). This is particularly relevant in anti-gamma-aminobutyric acid type B receptor (anti-GABA-B receptor) encephalitis, in which seizures often dominate and may delay recognition of an autoimmune limbic syndrome.

Anti-GABA-B receptor encephalitis is characterized by prominent seizures, cognitive or behavioral disturbance, and frequent limbic involvement (3–5), and small-cell lung cancer is the predominant malignancy among paraneoplastic cases (4–6). Although cases without detected malignancy may have better functional and survival outcomes (6), a tumor-negative status at presentation cannot be equated with definitive non-paraneoplastic disease, and repeated oncologic surveillance is recommended in high-risk settings (7).

Evidence gaps remain regarding bedside decision-making in severe, high-seizure-burden presentations. Anti-GABA-B receptor encephalitis is a recognized cause of new-onset refractory status epilepticus (NORSE), for which consensus recommendations support expedited etiologic evaluation and early immune-directed treatment once autoimmune encephalitis is suspected and infection reasonably excluded (8, 9). Therapeutic plasma exchange has been used in severe disease, but the evidence is observational and does not define optimal duration, sequencing, or anti-GABA-B-receptor-specific efficacy (10).

We report a 66-year-old man with anti-GABA-B receptor encephalitis and no malignancy on initial screening who developed refractory status epilepticus requiring mechanical ventilation and showed favorable short-term recovery during multimodal immune-directed and critical-care management. The report is deliberately bounded, illustrating a severe intensive care unit (ICU)-dependent presentation and the associated bedside decisions rather than a new phenotype or treatment principle.

## 2 Case presentation

**Figure 1:**
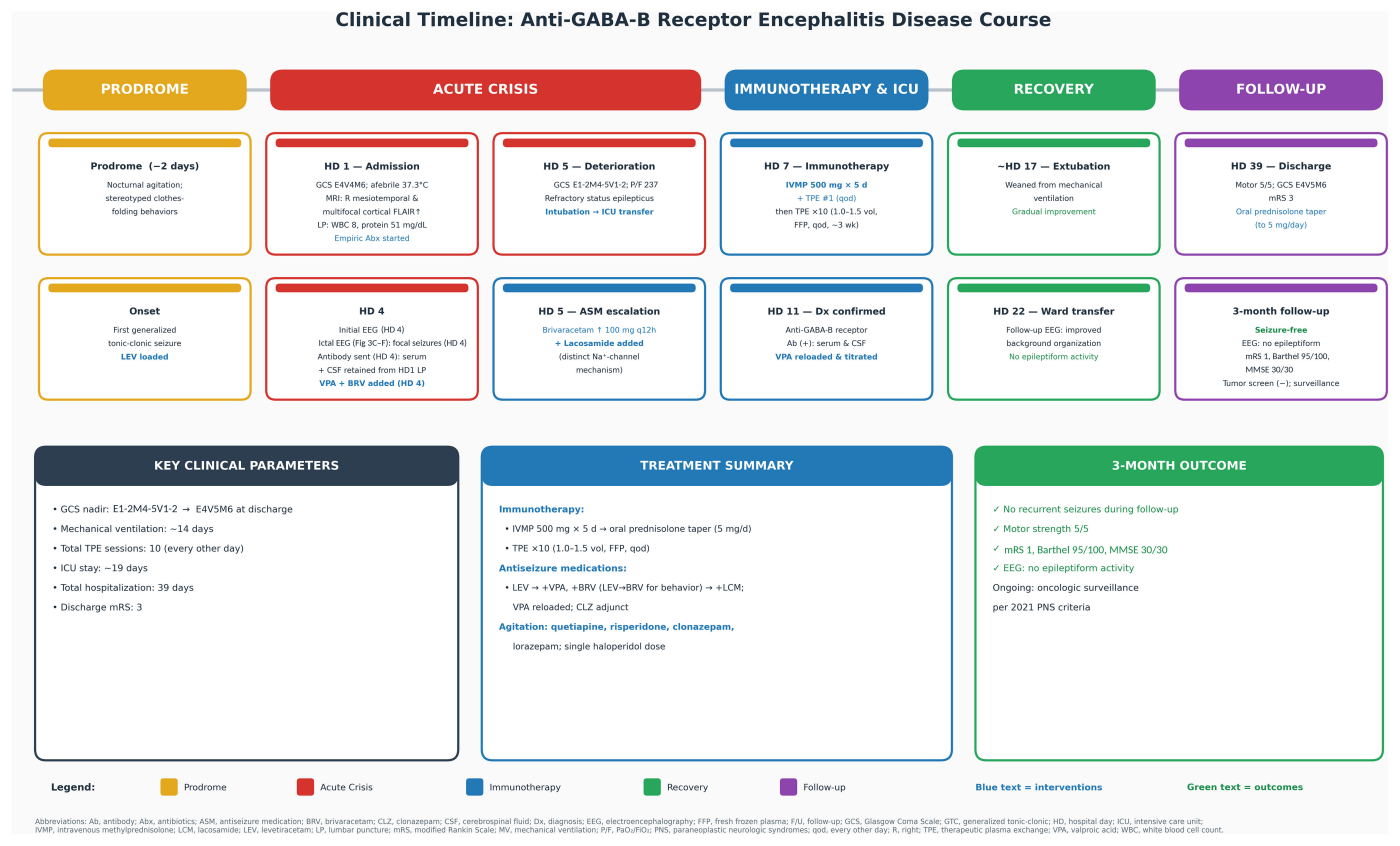
Clinical timeline of anti-GABA-B receptor encephalitis from the prodromal phase to 3-month follow-up. The schematic summarizes the behavioral prodrome, seizure onset, diagnostic work-up, intensive care unit course, immunotherapy, antiseizure medication escalation, symptomatic agitation management, extubation, discharge, and 3-month follow-up. Ab, antibody; Abx, antibiotics; ASM, antiseizure medication; BRV, brivaracetam; CLZ, clonazepam; CSF, cerebrospinal fluid; Dx, diagnosis; EEG, electroencephalography; F/U, follow-up; FFP, fresh frozen plasma; GCS, Glasgow Coma Scale; GTC, generalized tonic–clonic; HD, hospital day; ICU, intensive care unit; IV, intravenous; IVMP, intravenous methylprednisolone; LCM, lacosamide; LEV, levetiracetam; LP, lumbar puncture; mRS, modified Rankin Scale; MV, mechanical ventilation; P/F, PaO_2_/FiO_2_; PNS, paraneoplastic neurologic syndromes; qod, every other day; R, right; TPE, therapeutic plasma exchange; VPA, valproic acid; WBC, white blood cell count.

A 66-year-old man with hypertension, hyperlipidemia, and diet-controlled type 2 diabetes mellitus presented after a witnessed generalized tonic–clonic seizure lasting approximately 2 min, characterized by rightward gaze deviation, drooling, left lateral tongue biting, and limb clonic posturing. He was a non-smoker without alcohol or illicit substance use or significant occupational exposures. His family reported a 2-day history of nocturnal agitation and repetitive stereotyped clothes folding, followed by incoherent speech. These episodes occurred at home and were not captured on video EEG; in retrospect, they were suspicious for subtle focal seizures with automatisms but are reported only as a patient-specific clue. His history included a closed head injury in 2022 with complete recovery. A clinical timeline is shown in **Figure 1**.

At the outside hospital, the generalized seizure had self-terminated before medical contact, with intermittent left perioral twitching persisting on arrival. A 10-s convulsive recurrence noted in the emergency department was also self-terminated. No benzodiazepine was given before transfer, as the convulsive event had ended and the residual focal twitching was not judged to represent ongoing convulsive status epilepticus; intravenous levetiracetam 1,000 mg was loaded, and he was transferred to our tertiary center. On arrival, he was hemodynamically stable and afebrile (temperature 37.3 °C), with a Glasgow Coma Scale (GCS) score of E4V4M6. Neurologic examination showed paraphasia and disorientation to time and place, with preserved person orientation and intact global motor strength; meningeal signs were absent. A low-grade fever (up to 38.1 °C) developed over the first hospital days.

**Figure 2:**
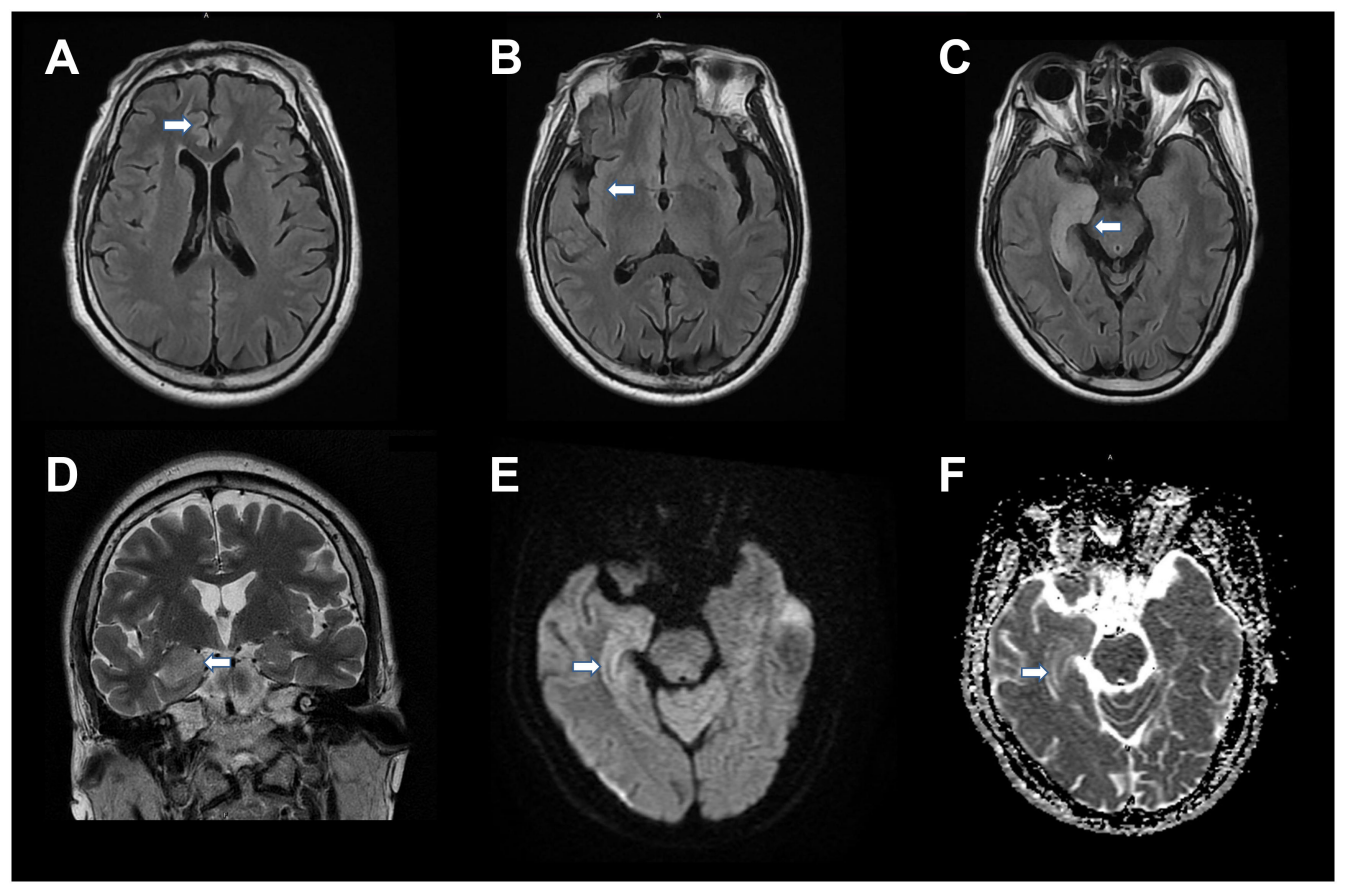
Admission magnetic resonance imaging showing right mesial temporal and multifocal cortical hyperintensities. Axial fluid-attenuated inversion recovery images demonstrate hyperintense signal in **(A)** the cingulate gyri and **(B)** the insular cortices, with right-sided predominance (arrows). **(C)** Axial fluid-attenuated inversion recovery image at the level of the mesial temporal lobe and **(D)** coronal T2-weighted image show right hippocampal and mesial temporal lobe hyperintensity (arrows). **(E)** Diffusion-weighted imaging shows apparent high signal in the right hippocampus (arrow); **(F)** the corresponding apparent diffusion coefficient map demonstrates elevated signal, consistent with T2 shine-through rather than true restricted diffusion (arrow).

Contrast-enhanced brain magnetic resonance imaging confirmed swelling and T2/fluid-attenuated inversion recovery (FLAIR) hyperintensity of the right hippocampus and mesial temporal lobe. Additional FLAIR hyperintensities involved the cingulate gyri and insular cortices, with right-sided predominance, consistent with multifocal cortical involvement (**Figure 2**). Diffusion-weighted imaging showed right hippocampal hyperintensity without corresponding apparent diffusion coefficient hypointensity. Lumbar puncture on hospital day 1 showed clear CSF with an opening pressure of 14.2 cm H_2_O, mild pleocytosis (white blood cell count 8 cells/μL), elevated protein 0.51 g/L (51 mg/dL), and glucose 3.3 mmol/L (60 mg/dL). The BIOFIRE FILMARRAY Meningitis/Encephalitis Panel (BioFire Diagnostics, Salt Lake City, UT, USA) was negative for common bacterial, viral, and fungal pathogens, including herpes simplex virus. Bacterial and fungal cultures showed no growth, and CSF cytology revealed no frank malignant cells.

**Figure 3:**
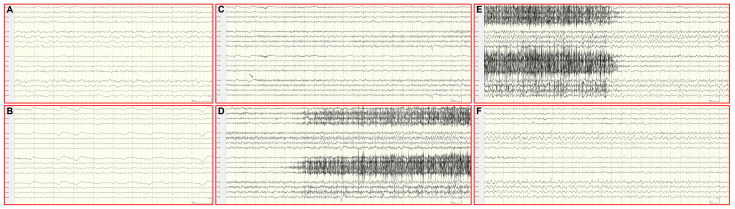
Initial and follow-up scalp electroencephalography demonstrating right hemispheric dysfunction and ictal fast activity (10–20 system, longitudinal bipolar montage, high-pass 1 Hz, low-pass 70 Hz, gain 7 μV/mm). **(A)** Interictal electroencephalography recorded on hospital day 4 showing a slow, disorganized right posterior dominant rhythm (5–7 Hz) and intermittent irregular slow activity over the right hemisphere. **(B)** Follow-up electroencephalography recorded on hospital day 13 demonstrating improved background organization. **(C)** Ictal onset recorded on hospital day 4 with focal paroxysmal fast activity in the beta range, maximal over the right frontal region (arrow). **(D–F)** Consecutive ictal epochs showing evolution of right hemispheric seizure activity with asymmetric muscle artifact reflecting associated clinical motor manifestations; bedside observation documented left-predominant clonic movements. Time-locked video was unavailable during the ictal recording.

After these self-limited convulsions, the patient returned to his neurologic baseline (GCS E4V4M6, full limb strength, and independent ambulation). Beginning on hospital day 4, recurrent focal motor seizures emerged. They initially presented as brief, seconds-long clonic twitching of the left lower limb. Later, recurrent perioral and facial twitching developed. The events increased in frequency over time. Between these events, the patient did not return to baseline: his GCS declined from E4V4M6 to E2M4V2 on hospital day 4 and to E1-2M4-5V1–2 on hospital day 5, with development of diffuse limb weakness. EEG on hospital day 4 showed a slow, disorganized right posterior dominant rhythm (5–7 Hz) and intermittent irregular slow activity over the right hemisphere (**Figure 3A**). Ictal onset was characterized by focal paroxysmal beta-range fast activity maximal over the right frontal region (**Figure 3C**), later evolving over the right hemisphere. Asymmetric muscle contractions were observed, more pronounced on the left side (**Figures 3D–F**), but ictal semiology could not be fully characterized because time-locked video was unavailable. These electrographic seizures persisted despite adequately dosed intravenous levetiracetam (1,500 mg twice daily) and valproate (400 mg every 8 h), supporting an operational diagnosis of refractory status epilepticus—ongoing focal electroclinical seizure activity despite two appropriately selected and adequately dosed intravenous antiseizure medications—and immediately preceded the clinical deterioration on hospital day 5 (9, 11).

Paired cerebrospinal fluid (from lumbar puncture on hospital day 1) and serum (obtained on hospital day 4) were sent to a contracted reference laboratory and assayed by cell-based indirect immunofluorescence (EUROIMMUN IIFT Autoimmune Encephalitis Mosaic 6; serum diluted 1:10, cerebrospinal fluid undiluted 1:1), which qualitatively detects IgG antibodies against the NMDA, AMPA1/AMPA2, GABA-B, LGI1, CASPR2, and DPPX antigens; a positive result was defined by the characteristic cell-surface (membrane) immunofluorescence pattern on the corresponding antigen-transfected cells, read against non-transfected control substrate. Anti-GABA-B receptor IgG was positive in both serum and CSF (reported on hospital day 11), whereas the remaining mosaic antibodies were negative; an end-point antibody titer was not determined. A comprehensive paraneoplastic onconeural antibody panel was also negative. Because the antibody mosaic used did not include the GABA-A receptor, anti-GABA-A receptor antibodies were not tested. Initial tumor screening with contrast-enhanced computed tomography (CT) of the chest, abdomen, and pelvis detected no malignancy, and serum tumor markers were within normal limits. Because occult malignancy may emerge only during longitudinal follow-up in anti-GABA-B receptor encephalitis, the patient was not considered definitively non-paraneoplastic; his malignancy status is therefore described as negative on initial screening, and structured oncologic surveillance was planned in accordance with the updated paraneoplastic neurologic syndrome recommendations (7).

The main differential diagnoses were viral meningoencephalitis, postictal MRI change, and autoimmune limbic encephalitis. Viral meningoencephalitis was excluded by negative multiplex pathogen testing and cultures, whereas focal-onset ictal EEG, multifocal cortical-mesiotemporal MRI abnormalities, and paired serum/CSF anti-GABA-B receptor positivity supported autoimmune encephalitis; postictal change alone could not explain the progressive behavioral and language disturbance, CSF inflammation, and antibody confirmation. Metabolic, toxic, systemic autoimmune, and thyroid-related causes were not supported by laboratory studies.

On hospital day 5, against this background of ongoing refractory status epilepticus, increased secretions, a gurgling voice, and declining oxygen saturation prompted arterial blood gas analysis, which showed acute hypoxemic respiratory failure (PaO_2_/FiO_2_ 237), likely aspiration-related. He was intubated for airway protection and transferred to the neurologic ICU. Laboratory testing showed neutrophilic leukocytosis with elevated C-reactive protein but low procalcitonin, supporting systemic inflammation without strong biochemical evidence of bacterial sepsis. Metabolic, thyroid, and systemic autoimmune testing was unrevealing.

Empirical ceftriaxone, vancomycin, and acyclovir had been started on hospital day 1, while infectious encephalitis was being excluded. On hospital day 7, after negative multiplex pathogen testing and cultures, empirical immunotherapy was initiated on syndromic grounds rather than antibody confirmation, consistent with recommendations that first-line immunotherapy should not be delayed pending neural-antibody results once infection is reasonably excluded (1, 2). Treatment included intravenous methylprednisolone 500 mg daily for 5 days, followed by an oral prednisolone taper to 5 mg daily by discharge. Therapeutic plasma exchange was initiated on hospital day 7 and performed every other day, with each session exchanging 1.0–1.5 plasma volumes. Fresh frozen plasma was used as the replacement fluid in accordance with institutional apheresis practice. This approach was chosen to replace coagulation factors, maintain fibrinogen during the intensive alternate-day schedule, and reduce the risk of dilutional coagulopathy; we recognize that 5% albumin is the more commonly used replacement fluid and carries a lower risk of allergic and infectious reactions (12, 13). The number of plasma exchange sessions did not follow a fixed protocol. Continuation was guided mainly by the Glasgow Coma Scale, behavioral status, and cognitive recovery. Persistent electrographic seizures were not the reason for continuing plasma exchange because continuous EEG monitoring showed no sustained ictal activity. After extubation, the remaining exchanges were continued because behavioral and cognitive recovery remained incomplete, reaching 10 sessions over 3 weeks (12). Antibiotics were escalated to meropenem for suspected aspiration pneumonia.

Antiseizure management was adjusted stepwise according to the electroclinical course and tolerability; levetiracetam was the initial agent. When focal seizures persisted on hospital day 4, valproic acid (400 mg every 8 h) was added as a broad-spectrum agent, and levetiracetam was switched to brivaracetam (50 mg every 12 h) to maintain SV2A-targeted therapy while reducing concern for levetiracetam-associated behavioral effects (14). On hospital day 5, with persistent facial twitching, focal right-hemispheric ictal activity, and progression to refractory status epilepticus, brivaracetam was increased to 100 mg every 12 h, and lacosamide (100 mg every 12 h) was added for its distinct sodium channel mechanism. Valproic acid was reloaded (800 mg) during an exacerbation on hospital day 11 and titrated to 600 mg every 8 h after a subtherapeutic level, with clonazepam as adjunctive therapy. This escalation was individualized, recognizing that antiseizure drug selection in autoimmune encephalitis remains incompletely standardized (15). The discharge regimen comprised valproic acid 800 mg three times daily, brivaracetam 100 mg twice daily, lacosamide 50 mg twice daily, and clonazepam 1 mg nightly.

Continuous anesthetic infusion was not required for seizure control. Midazolam was used only briefly for post-intubation sedation (hospital days 5 and 6) and for procedural sedation during plasma exchange sessions when agitation precluded cooperation, with propofol substituted once; each infusion was discontinued immediately after the session. Electroencephalographic monitoring showed no sustained ictal activity throughout the intensive care course, and the presentation did not escalate to super-refractory status epilepticus.

Agitation was multifactorial (active limbic encephalitis, seizures, mechanical ventilation, ICU delirium, medication effects, and procedural intolerance), and risperidone alone was initially insufficient. Scheduled quetiapine and clonazepam were introduced on hospital day 6, oral lorazepam (0.5 mg three times daily) and risperidone (1 mg twice daily) on hospital day 9, and a single dose of haloperidol (2.5 mg) on hospital day 11, supplemented by short-acting procedural sedation and temporary physical restraint for airway and line protection. Behavioral symptoms gradually abated as immunotherapy progressed, and risperidone was tapered after stabilization.

The patient improved gradually. A follow-up EEG on hospital day 13 showed resolution of slow activity and improved right hemispheric background organization (**Figure 3B**). He was extubated after approximately 2 weeks and transferred to the general ward on hospital day 22. At discharge on hospital day 39, strength had normalized (5/5 throughout), the GCS was E4V5M6, and he was alert and fully oriented, although complex instrumental activities of daily living remained impaired (modified Rankin Scale score 3). At 3-month follow-up, he remained free of recurrent clinical seizures, and behavioral symptoms had resolved. Functional recovery was substantial and objectively documented: the modified Rankin Scale score had improved from 3 at discharge to 1, the Barthel Index for activities of daily living was 95 of 100, and the Mini-Mental State Examination score was 30 of 30. He was maintained on three antiseizure medications—lacosamide 50 mg twice daily, brivaracetam 100 mg twice daily, and valproic acid 600 mg three times daily—after down-titration of valproic acid and discontinuation of clonazepam from the discharge regimen; antipsychotic therapy for intensive care agitation had been reduced to low-dose as-needed quetiapine (12.5 mg nightly), which was being tapered. Renal and hepatic function remained normal. To date, no repeat chest CT or whole-body positron emission tomography–computed tomography (PET–CT) has been performed; during the 3-month follow-up, oncologic surveillance has consisted of interval chest radiographs, which have remained unremarkable. Structured surveillance is planned at 4–6-month intervals for at least 2 years, prioritizing chest-focused imaging given the predominance of small-cell lung cancer in paraneoplastic disease (4, 6). Whole-body [^18^F]fluorodeoxyglucose PET–CT (unavailable during the acute admission) is under consideration for a subsequent evaluation and has been discussed with the patient and family; its use after an unrevealing thoracic CT is consistent with prior tumor-screening recommendations (16) and complements the repeat conventional imaging advised by updated paraneoplastic neurologic syndrome criteria (7).

## 3 Discussion

The individual features of this case—seizure-predominant onset, neuropsychiatric disturbance, ICU-level severity, progression to refractory status epilepticus, malignancy association, and recovery after immune-directed treatment—have all been described previously (3–5, 17); its value lies in their combination in one ventilator-dependent patient with paired serum/CSF confirmation and no malignancy on initial screening. Notably, the multifocal cortical FLAIR hyperintensity extending into the cingulate gyri and insular cortices is reported more characteristically as a core imaging feature of anti-GABA-A receptor encephalitis (18) than of anti-GABA-B receptor encephalitis, in which it occurs in a minority of cases (~22%) (4). Because the antibody mosaic used did not include the GABA-A receptor, anti-GABA-A receptor antibodies were not tested; although the unequivocal presence of anti-GABA-B receptor IgG in both serum and cerebrospinal fluid supports the diagnosis, a coexisting or alternative anti-GABA-A receptor reactivity cannot be excluded and is acknowledged as a limitation.

The nocturnal repetitive behavior was retrospectively suspicious for subtle focal seizures with automatisms, particularly given the right hippocampal and mesial temporal abnormalities, but was not captured on video EEG and should be treated as a patient-specific recognition clue rather than a generalizable marker; larger cohorts have shown that isolated seizures may precede the encephalitic phase, especially in paraneoplastic disease (17).

The case also illustrates the acute dilemma of distinguishing infectious meningoencephalitis from autoimmune limbic encephalitis before antibody results are available. Subacute behavioral and language disturbance, low-grade fever, mild CSF pleocytosis, mesiotemporal MRI abnormalities, and acute seizures justified empirical antimicrobial therapy, while herpes simplex and other treatable infections were excluded (1, 19); progression to refractory status epilepticus, focal ictal EEG, and negative infectious testing then shifted probability toward autoimmune encephalitis and supported immunotherapy before antibody confirmation. The discriminative tool of Moser and colleagues formalizes this dilemma, showing that routine CSF, imaging, and clinical features may be insufficient to distinguish autoimmune from infectious encephalitis acutely (20).

Several management decisions are instructive. First, empirical immunotherapy was based on syndrome recognition rather than antibody confirmation: samples were sent on hospital day 4, but the anti-GABA-B receptor result returned only on hospital day 11, after immunotherapy had begun on hospital day 7; in hindsight, this threshold may have been reached earlier once infectious testing was negative, although whether earlier treatment would have changed the course is unknown; observational data suggest that earlier seizure freedom after immunotherapy than with antiseizure medications alone (21). Second, antiseizure and behavioral treatment required repeated reassessment because agitation could reflect active encephalitis, recurrent seizures, ICU delirium, medication effects, or procedural intolerance; the regimen was individualized rather than algorithmic—valproic acid, lacosamide, and brivaracetam, with adjunctive benzodiazepines and antipsychotics—and drug selection, duration, and withdrawal in autoimmune encephalitis remain insufficiently standardized and should be individualized alongside immunotherapy (15); super-refractory status epilepticus was avoided. Third, the 10-session plasma exchange course should be interpreted cautiously: no guideline defines its optimal duration in anti-GABA-B receptor encephalitis, and the evidence is heterogeneous and largely non-randomized (10); because plasma exchange was one component of a multimodal rescue strategy, the temporal association between treatment and recovery cannot establish its independent benefit. Fourth, paraneoplastic risk stratification remained incomplete: KCTD16 autoantibodies, associated with malignancy risk, were unavailable at our institution (3, 6), so negative initial CT and tumor markers cannot exclude occult malignancy; updated criteria support repeat oncologic screening over time, and small-cell lung cancer may be diagnosed only after repeated evaluation (7, 22).

This single uncontrolled case has only 3-month follow-up, and KCTD16 testing, antibody titers, an intrathecal immunoglobulin G index, and acute-phase PET–CT were unavailable; the favorable short-term course cannot be generalized to long-term prognosis or used to infer treatment efficacy. Nevertheless, the case reinforces that adult NORSE is a clinical presentation rather than a final diagnosis and should prompt expedited evaluation for autoimmune encephalitis once acute structural, toxic, metabolic, and infectious causes are not immediately identified (8, 9).

## 4 Conclusion

This case illustrates severe anti-GABA-B receptor encephalitis presenting with refractory status epilepticus and no malignancy detected on initial screening, followed by favorable short-term recovery during multimodal immune-directed and intensive care management. The report is hypothesis-generating and does not establish treatment efficacy, optimal plasma exchange duration, long-term prognosis, or definitive non-paraneoplastic status. Within these limits, the main lessons are that new-onset refractory status epilepticus should trigger expedited autoimmune encephalitis evaluation, that nocturnal stereotyped behavior may be a patient-specific clue to focal seizures, that plasma exchange may be considered as one component of multimodal rescue in selected severe cases, and that structured oncologic surveillance remains essential despite negative initial tumor screening.

## 5 Patient perspective

From the perspective of the patient and his family, the most distressing aspect of the intensive care course was that he became confused and did not recognize them. The medical team’s explanation that these abrupt behavioral and cognitive changes were manifestations of autoimmune encephalitis helped them understand the clinical course and the rationale for empirical antimicrobial and immune-directed therapy before the diagnosis was confirmed. At 3-month follow-up, the patient and family reported a meaningful recovery in daily activities and emphasized the importance of antiseizure treatment adherence and continued malignancy surveillance. They also reflected that the nocturnal behavioral changes preceding the convulsion had initially been underestimated.

## Data Availability

The original contributions presented in the study are included in the article/**Supplementary Material**. Further inquiries can be directed to the corresponding author.
