# Supplementary material for: Case Report: Anti-GABA-B receptor encephalitis with refractory status epilepticus and favorable short-term recovery
Source: Front Immunol. 2026 Aug 4;17:1896400. doi: 10.3389/fimmu.2026.1896400 (PMC13481455; doi:10.3389/fimmu.2026.1896400)
Supplement: Supplementary file 1 [file Table1.docx]

**Supplementary Material: CARE Checklist (2016) of Information to Include When Writing a Case Report**

**Manuscript:** “Case Report: Anti-GABA-B Receptor Encephalitis With Refractory Status Epilepticus and Favorable Short-Term Recovery” (submitted to Frontiers in Immunology, Case Report).

**Instructions:** The CARE checklist is completed by referencing every item against the section of the manuscript in which it is reported. Location references correspond to the integrated final manuscript. Where line numbers are required by the journal, the corresponding-author should insert continuous line numbers and update the locations accordingly.

| **No.** | **CARE Checklist Item** | **Reported** | **Section / Location in Manuscript** |
| --- | --- | --- | --- |
| 1 | Title — The words “case report” appear in the title, along with the phenomenon of greatest interest. | Yes | Title: “Case Report: Anti-GABA-B Receptor Encephalitis With Refractory Status Epilepticus and Favorable Short-Term Recovery.” |
| 2 | Key Words — 2 to 5 key words that identify topics in this case report. | Yes | Keywords (after author block): case report; anti-GABA-B receptor encephalitis; autoimmune encephalitis; refractory status epilepticus; therapeutic plasma exchange |
| 3a | Abstract — Introduction: What is unique and why is it important? | Yes | Abstract, sentence 1 and final 3 sentences (rarity of anti-GABA-B receptor encephalitis; bounded, hypothesis-generating contribution). |
| 3b | Abstract — Main symptoms / clinical findings; main diagnoses, interventions, outcomes. | Yes | Abstract, sentences 2–7 (presentation, MRI/CSF findings, RSE, antibody confirmation, treatment, discharge mRS 3, 3-month outcome). |
| 3c | Abstract — Conclusion / take-away lessons. | Yes | Abstract, final 2 sentences (hypothesis-generating limits; NORSE → AE work-up; oncologic surveillance). |
| 4 | Introduction — Brief background summary referencing relevant medical literature. | Yes | Introduction (4 paragraphs), references (1–5, 7–9, 12, 16). |
| 5a | Patient Information — De-identified demographic and other patient information. | Yes | Case Presentation, paragraph 1 (66-year-old man; hypertension, hyperlipidemia, diet-controlled type 2 diabetes; non-smoker; closed head injury 2022). |
| 5b | Patient Information — Main concerns and symptoms of the patient. | Yes | Case Presentation, paragraph 1 (witnessed generalized tonic-clonic seizure; 2-day nocturnal repetitive stereotyped behaviors; incoherent speech). |
| 5c | Patient Information — Medical, family, and psychosocial history including relevant genetic information. | Yes | Case Presentation, paragraph 1 (comorbidities, social history, prior closed head injury). No relevant family/genetic history identified; KCTD16 testing unavailable (noted in Discussion). |
| 5d | Patient Information — Relevant past interventions and their outcomes. | Yes | Case Presentation, paragraph 1–2 (closed head injury 2022 with complete recovery; outside-hospital intravenous levetiracetam loading). |
| 6 | Clinical Findings — Describe the relevant physical examination and other significant clinical findings. | Yes | Case Presentation, paragraph 2–3 (vital signs, GCS E4V4M6, paraphasia, disorientation, intact motor strength, absent meningeal signs; MRI, CSF, EEG findings). |
| 7 | Timeline — Important dates and times in this episode of care are organized into a timeline (figure or table). | Yes | Figure 1 (clinical timeline from prodrome to 3-month follow-up); cited in Case Presentation, paragraph 1. Hospital days 1–39 and 3-month follow-up referenced throughout Case Presentation. |
| 8a | Diagnostic Assessment — Diagnostic methods (e.g., PE, laboratory testing, imaging, questionnaires). | Yes | Case Presentation, paragraphs 3–5 (contrast-enhanced brain MRI; lumbar puncture with opening pressure, cell count, protein, glucose; BIOFIRE FILMARRAY ME panel; cultures; cytology; EEG; paired serum/CSF cell-based antibody assays; onconeural panel; contrast-enhanced CT chest/abdomen/pelvis; serum tumor markers). |
| 8b | Diagnostic Assessment — Diagnostic challenges (e.g., access, financial, cultural). | Yes | Case Presentation, paragraph 5 and Discussion, paragraph 3–4 (antibody assays not point-of-care; result returned hospital day 11; KCTD16 testing unavailable at institution; infectious-vs-autoimmune diagnostic overlap). |
| 8c | Diagnostic Assessment — Diagnostic reasoning including other diagnoses considered. | Yes | Case Presentation, paragraph 6 (differential diagnoses: viral meningoencephalitis, postictal MRI change, autoimmune limbic encephalitis; metabolic/toxic/thyroid causes excluded). |
| 8d | Diagnostic Assessment — Prognostic characteristics (e.g., staging) where applicable. | Yes | Case Presentation, paragraphs 5 and 9, and Discussion, paragraphs 1 and 4 (no malignancy detected on initial screening; malignancy status considered provisional; ICU-dependent disease and structured oncologic surveillance discussed). |
| 9a | Therapeutic Intervention — Types of intervention (e.g., pharmacologic, surgical, preventive, self-care). | Yes | Case Presentation, paragraph 8 (intravenous methylprednisolone; therapeutic plasma exchange; multi-agent antiseizure therapy; empirical antimicrobials; mechanical ventilation; risperidone). |
| 9b | Therapeutic Intervention — Administration of intervention (e.g., dosage, strength, duration). | Yes | Case Presentation, paragraph 8 (IVMP 1000 mg/day × 3 days then oral taper to 4 mg/day; TPE every other day × 10 sessions over 3 weeks, 1.0–1.5 plasma volumes, FFP replacement; discharge ASM regimen with specific doses). |
| 9c | Therapeutic Intervention — Changes in intervention (with rationale). | Yes | Case Presentation, paragraph 8 and Discussion, paragraph 4 (levetiracetam → brivaracetam for suspected agitation; antibiotic escalation to meropenem for aspiration pneumonia; pragmatic selection of extended 10-session TPE). |
| 10a | Follow-up and Outcomes — Clinician- and patient-assessed outcomes (when appropriate). | Yes | Case Presentation, paragraph 9 (extubation ~2 weeks; ward transfer day 22; discharge day 39 with mRS 3, GCS E4V5M6; 3-month seizure freedom, improving function, EEG without epileptiform discharges). |
| 10b | Follow-up and Outcomes — Important follow-up diagnostic and other test results. | Yes | Case Presentation, paragraph 9 (follow-up EEG improvement; 3-month EEG without epileptiform discharges; mild anemia [Hb 92 g/L]; normal renal/hepatic function); ongoing oncologic surveillance every 4–6 months. |
| 10c | Follow-up and Outcomes — Intervention adherence and tolerability (how was this assessed). | Yes | Case Presentation, paragraph 8–9 (no major TPE-related adverse events; risperidone tapered after resolution of behavioral symptoms; emphasis on antiseizure-treatment adherence — see Patient Perspective). |
| 10d | Follow-up and Outcomes — Adverse and unanticipated events. | Yes | Case Presentation, paragraph 5, 7–8 (aspiration-related hypoxemic respiratory failure requiring intubation; suspected levetiracetam-associated agitation; suspected aspiration pneumonia; no major procedure-related adverse events from TPE). |
| 11a | Discussion — Strengths and limitations in the management of this case. | Yes | Discussion, paragraph 3–5 (bedside decision-making strengths; limitations: single uncontrolled case, 3-month follow-up, unavailable KCTD16/titers/IgG index/PET-CT). |
| 11b | Discussion — Relevant medical literature (similar and contrasting cases). | Yes | Discussion, paragraphs 1–4 (comparison with prior cohorts and case series; anti-GABA-A vs anti-GABA-B imaging; paraneoplastic vs nonparaneoplastic outcomes); references (3–16). |
| 11c | Discussion — Rationale for conclusions (including assessment of cause and effect). | Yes | Discussion, paragraph 4–5 and Conclusion (favorable course interpreted as temporal association with multimodal rescue; explicit non-attribution of efficacy to any single therapy). |
| 11d | Discussion — Primary take-away lessons of this case report. | Yes | Conclusion (NORSE → expedited AE work-up; nocturnal stereotyped behavior as patient-specific clue; TPE as multimodal-rescue component; structured oncologic surveillance despite negative initial screening). |
| 12 | Patient Perspective — The patient should share their perspective on the treatment(s) they received. | Yes | Patient Perspective section (family distress during ICU course; value of clinician communication; meaningful 3-month recovery; recognition of underestimated nocturnal prodrome). |
| 13 | Informed Consent — Did the patient give informed consent? Provide if requested. | Yes | Ethics Statement (written informed consent obtained from the patient’s son (next of kin) for publication, including accompanying images; IRB approval, Tri-Service General Hospital). Yes — available on request. |

Checklist completed by: Fu-Chi Yang (corresponding author), on behalf of all authors.

Date: _11-July-2026_

Reference: Gagnier JJ, Kienle G, Altman DG, Moher D, Sox H, Riley D, et al. The CARE guidelines: consensus-based clinical case reporting guideline development. J Clin Epidemiol. (2014) 67:46–51. doi: 10.1016/j.jclinepi.2013.08.003
